# Social patterning of childhood overweight in the French national ELFE cohort

**DOI:** 10.1038/s41598-023-48431-8

**Published:** 2023-12-11

**Authors:** Camille Le Gal, Marion Lecorguillé, Lorraine Poncet, Aminata Hallimat Cissé, Malamine Gassama, Thierry Simeon, Jean-Louis Lanoë, Maria Melchior, Jonathan Y. Bernard, Marie-Aline Charles, Barbara Heude, Sandrine Lioret

**Affiliations:** 1 Université Paris Cité and Université Sorbonne Paris Nord, Inserm, INRAE, Center for Research in Epidemiology and StatisticS (CRESS), 75004 Paris, France; 2 UMS INED-INSERM-EFS, Paris, France; 3 IPLESP, INSERM U1136, Paris, France

**Keywords:** Risk factors, Public health, Weight management, Epidemiology

## Abstract

An inverse social gradient in early childhood overweight has been consistently described in high-income countries; however, less is known about the role of migration status. We studied the social patterning of overweight in preschool children according to the mother’s socio-economic and migration background. For 9250 children of the French ELFE birth cohort with body mass index collected at age 3.5 years, we used nested logistic regression to investigate the association of overweight status in children with maternal educational level, occupation, household income and migration status. Overall, 8.3% (95%CI [7.7–9.0]) of children were classified as overweight. The odds of overweight was increased for children from immigrant mothers (OR 2.22 [95% CI 1.75–2.78]) and descendants of immigrant mothers (OR 1.35 [1.04–2.78]) versus non-immigrant mothers. The highest odds of overweight was also observed in children whose mothers had low education, were unemployed or students, or were from households in the lowest income quintile. Our findings confirm that socio-economic disadvantage and migration status are risk factors for childhood overweight. However, the social patterning of overweight did not apply uniformly to all variables. These new and comprehensive insights should inform future public health interventions aimed at tackling social inequalities in childhood overweight.

## Introduction

The high prevalence of overweight and obesity, estimated at 17.9% in European children aged 2 to 7 years^1^ and its social patterning^2^ are a major public health issue. Several studies have identified an inverse social gradient of childhood overweight in high-income countries; children from families with low socioeconomic position (SEP) seem more affected. Excess weight is associated with many harmful comorbidities in children, both physical and mental (e.g. hypertension, type 2 diabetes mellitus, dyslipidemia, and psychological distress)^3^ and increases the risk of obesity in adulthood, thus contributing to social inequalities in health throughout life^4^.

Factors contributing to excess weight are multifaceted, involving a complex interplay between genetic predisposition and environmental^5^, social and behavioral determinants, which operate at different levels^6^. One of these determinants is children’s or family SEP, which is commonly characterized by various indicators in the literature, such as parents’ educational level, occupation, or household income. Although correlated, these indicators are all important to be considered because they capture not–inter-changeable dimensions of SEP^7^. For example, parents’ educational level is indicative of their knowledge and, to some extent, their health literacy and skills; parents’ occupational category provides insight into the social environment in which the child is raised; and household income reflects the family’s purchasing capacity and ability to access goods and services^7^. Of note, maternal education was found the strongest predictor of child overweight, which can be attributed in part to the temporal consistency of maternal educational level, in contrast to the variability of occupation and income^8^. A study of 11 European cohorts showed that as early as preschool, children whose mothers had a lower degree of education were more likely to be overweight than those whose mothers had a higher educational degree. Still, parental occupation and household income were also found associated with child overweight and should deserve equal attention^9^. The few studies that focus on these multiple indicators usually do not cautiously address their interrelation, especially when introducing them all at once in multi-adjusted models, which impairs the interpretation of the results. Davison et al.^10^ recommended ordering the various determinants of childhood overweight according to their proximity to the outcome, under a socio-ecological conceptual framework. Accordingly, we hypothesized that educational level would be an upstream factor, followed by occupational category, which would in turn influence income.

Beyond these common socio-economic factors, the socio-cultural determinants of child overweight have been under-studied and are worth considering for addressing the issue of inequalities more comprehensively. In particular, people with an immigration background are disproportionally affected by overweight and obesity^11–13^. To date, European data on the health of immigrants and their descendants remain partial and results are inconsistent^14^. According to the “healthy migrant effect”, individuals who are able to migrate have a better health status on average than their non-migrating counterparts, both in their country of birth and in the host country^15^. However, the health status for ethnic/racial minorities deteriorates once they have settled in the host country. This deterioration increases over time, mainly due to poor working and housing conditions, downward social mobility, and difficulties in accessing care^5,16^, along with an acculturation process, which can affect individuals’ health behaviours. In this context and as suggested by Jusot et al.^17^, migration status may be at the most distal position in our conceptual framework, upstream of the other SEP indicators.

This study investigated the early social patterning of overweight in preschool children while accounting for both mothers’ SEP and migration status in the ELFE national birth cohort.

## Material and methods

### Study population

The ELFE study is a nationwide, multidisciplinary birth cohort^18^. It recruited 18,329 children born in a random sample of 344 maternity units located in metropolitan France over 25 days in four different periods in 2011. Inclusion criteria were single or twin births ≥ 33 weeks of amenorrhea and mothers ≥ 18 years old and not planning to leave *Mainland* France within 3 years. To ensure the inclusion of women with limited French literacy, the information letter and consent form were translated into Arabic, Turkish, and English, the three most commonly spoken languages in France in non-French speakers. Among the eligible mothers, 51% agreed to participate in the study and provided informed and written consent for their and their child’s participation. Fathers gave signed consent for the child’s participation when present at inclusion or were informed about their rights to oppose it. The ELFE study received approvals from the Advisory Committee for the Processing of Information for Health Research (Comité Consultatif sur le Traitement des Informations pour la Recherche en Santé) and National Data Protection Authority (Commission National Informatique et Libertés) and the National Council for Statistical Information. All research was performed in accordance with the Declaration of Helsinki.

### Data collection and measurements

Data collection included a comprehensive set of information on maternal and paternal characteristics, pregnancy, birth, and postnatal outcomes as well as follow-up data on the child’s health, development, and environment.

Data collection methods included a face-to-face interview conducted by trained investigators at the maternity hospital and computer-assisted telephone interviews conducted by a survey institute at the 2-month, 2-year and 3.5-year follow-ups. In addition, information from the medical record, including the mother’s health history and the child’s anthropometric measurements and health status at birth, was collected at the maternity hospital. Information on the mother’s SEP and migration history was collected during the 2-month follow-up.

During the telephone follow-up interview at 3.5 years, parents were asked to report their child’s weight and height from the health booklet. From these, the body mass index (BMI) was calculated for each child (weight [kg]/[height (m)]^2^), as was overweight (including obesity) defined using the International Obesity Task Force (IOTF)^19^ age- and sex-specific reference (BMI ≥ IOTF-25 cut-off).

Maternal migration status was defined in three categories: “non-immigrant” for women born French to two French parents (in or outside of France); “descendant of immigrant” for women born in France to at least one non-French parent; and “immigrant” for women born outside France, and without French nationality at birth. Additionally, the maternal country of birth was used as a descriptive variable to further characterize the migration dimension. The variable was classified as follows: France, European Union (except France), Turkey and North Africa, Sub-Saharan Africa, and other (other Africa, Eastern Europe, Asia, America).

Three indicators of SEP were defined in categories as follows: maternal educational level (≤ high school level, 1–2 years university degree, and ≥ 3-year university degree); maternal occupation (“executive and top management”, “middle occupation [teacher, nurse, technician, foreman]”, “farmer and skilled blue-collar worker”, “clerk”, “manual worker, unemployed and student “); and household income level per consumption unit, categorized into quintiles. According to the definition provided by the French National Institute of Statistics and Economic Studies, the latter indicator is calculated by allocating distinct weights to each member of a household, depending on the member’s age (1 consumption unit for the householder, 0.5 for other household members aged 14 or over, and 0.3 for each child aged less than 14 years). This enables the comparison of living standards across households with varying sizes and compositions^20^.

The adjustment variables considered for this study included child sex, maternal age at delivery (in years), and parity (first-time mother, second-time mother or multiparous).

### Study sample selected for statistical analyses

**Figure 1:**
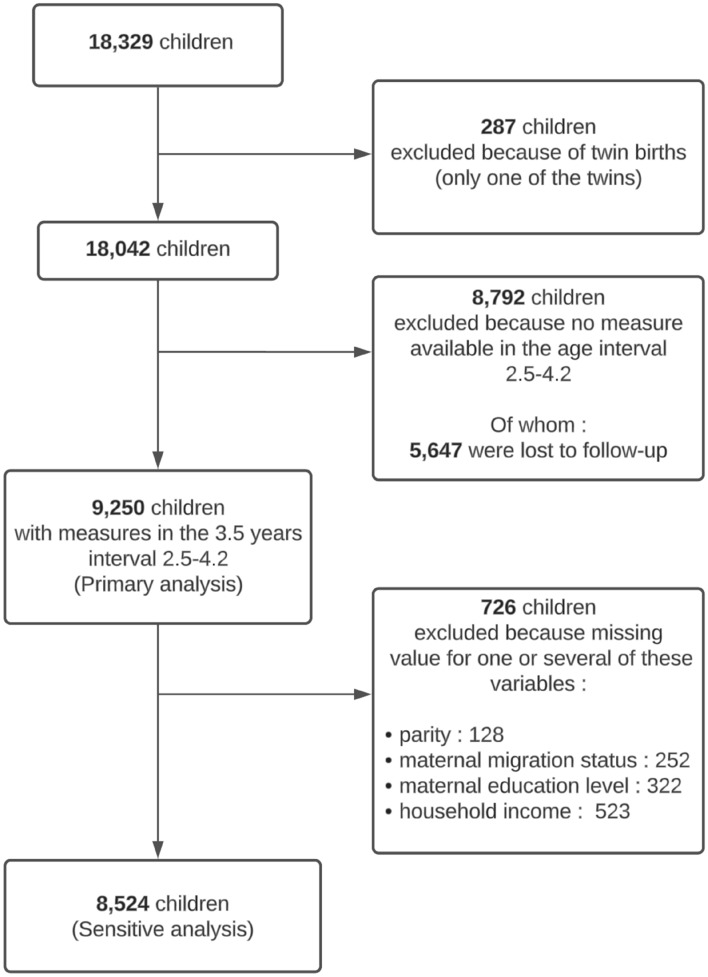
Flow chart of the study sample.

For twin births, the study inclusion was limited to one twin chosen randomly (Fig. 1). Measurements were selected from an age range of 2.5 to 4.2 years, with a mean child age of 3.5 years. Only children with at least one BMI measure available within this age range were eligible for analysis (N = 9250). When multiple BMI measures were available for the same child, only the last one was used.

### Statistical analyses

We first estimated the prevalence of children’s overweight at 3.5 years, before and after accounting for the study design and non-response at inclusion and at follow-up, using specific weighting. This weighting method included calibration on margins from the state register’s statistical data as well as data from the 2010 French National Perinatal study^21^ for the following variables: sex, region, parity, marital status, mother’s and father’s age, mother’s education, mother’s migration status, mother’s and father’s employment at the time of delivery, child-birth preparation sessions, mother living in a partnership at birth, and alcohol consumption during pregnancy. This approach complies with the procedures recommended by the French National Institute of Statistics and Economic Studies.

**Figure 2:**
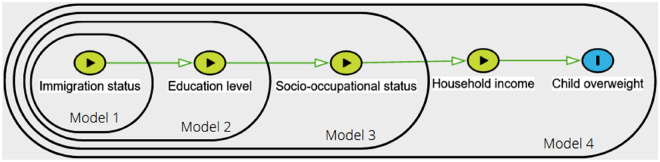
Directed acyclic graph and the four nested variables based on our hypothesized conceptual framework driving the statistical analysis strategy.

The associations of overweight status in children aged 3.5 years with maternal migration status and SEP indicators (maternal educational level, maternal occupational category and household income) were first assessed using weighted chi-squared tests. These migration and SEP indicators were structured from the most distal to the most proximal within a four–nested-variable framework, derived from both socio-ecological^10^ and hierarchical approaches^22^. As stated in the introduction, we assumed that migration status and educational level were at the most distal levels, with the latter influencing occupational category, which in turn would impact income level, as the most proximal variable^7^. Accordingly, model 1 included maternal migration status. Models 2, 3 and 4 successively added maternal educational level, maternal occupational category and household income (Fig. 2). This hierarchical approach was intended to ensure that intermediate SEP variables did not affect the association of the more distal migration and SEP indicators with the outcome under study (i.e., overweight status). Consequently, the results for each variable are interpreted as they are added to the model. Unweighted logistic regression models were used to assess the odds of child overweight according to variations in migration or SEP modalities, estimating odds ratios (ORs) and 95% confidence intervals (CIs). All four models were adjusted for child sex, maternal age at delivery and parity. Missing values were imputed using the missForest R package, which uses random forest imputation techniques. The random forest approach, introduced by Breiman in 2001^23^, is a highly effective method for both classification and regression. By combining multiple randomized decision trees and averaging their predictions, this algorithm has demonstrated very good performance^24^. Furthermore, to ascertain the necessity of stratification, we tested for interactions between maternal migration status and each SEP variable by using an alpha risk threshold of 0.05.

Primary multivariable analyses were conducted with imputation of missing values for parity, maternal migration status, maternal educational level and household income. Sensitivity analyses were conducted for complete cases (n = 8524).

All statistical analyses involved using RStudio (2022) (RStudio: Integrated Development Environment for R. RStudio, PBC, Boston, MA).

## Results

Of the 9250 children included in the study, 9.8% and 8.5% were born to mothers who were descendants of immigrants or immigrants, respectively (Table 1). Most mothers (90.3%) were born in France, whereas those born outside of France were mainly from North Africa and Turkey (3.3%) and Sub-Saharan Africa (2.4%). Overall, 43.5% of mothers had ≥ 3-year university education and 32.5% had ≤ high school education; 43.5% were employed and the median monthly household income was €1619 (IQR 1238–2048) per consumption unit.

**Table 1:** Socio-demographic and socio-economic characteristics of mothers and prevalence of overweight status for children aged 3.5 years in the national ELFE cohort (unweighted data).

	Overall N = 9250	Not overweight N = 8 597 92.9%	Overweight N = 653 7.1%
Sex of the child			
Girl	48.9% (4 519)	48.5% (4 170)	53.4% (349)
Maternal age at delivery (years)	31.0 (28.0–35.0)	31.0 (28.0–34.0)	31.0 (28.0–35.0)
Parity			
First-time mother	48.1% (4 386)	48.7% (4 129)	40.0% (257)
Second-time mother	35.9% (3 279)	35.7% (3 027)	39.2% (252)
Third-time mother	16.0% (1 457)	15.6% (1 323)	20.8% (134)
Missing values	128	118	10
Single motherhood			
Yes	2.5% (221)	2.4% (197)	3.9% (24)
Missing values	279	246	33
Maternal migration status			
Non-immigrant	81.7% (7 355)	82.4% (6 904)	72.3% (451)
Descendant of immigrant	9.8% (878)	9.6% (805)	11.7% (73)
Immigrant	8.5% (765)	7.9% (665)	16.0% (100)
Missing values	252	223	29
Maternal country of birth			
France	90.3% (8 347)	90.8% (7 802)	83.5% (545)
European Union	2.3% (213)	2.3% (198)	2.3% (15)
Turkey and Maghreb	3.3% (301)	3.0% (257)	6.7% (44)
Sub-Saharan Africa	2.4% (222)	2.2% (193)	4.4% (29)
Other	1.8% (164)	1.7% (144)	3.1% (20)
Missing values	3	3	0
Maternal education level			
≥ 3-year university	43.5% (3 886)	44.3% (3 677)	33.6% (209)
1- to 2-year university	24.0% (2 142)	24.0% (1 993)	24.0% (149)
≤ High school	32.5% (2 900)	31.7% (2 636)	42.4% (264)
Missing values	322	291	31
Maternal occupational category			
Executive and top management	21.1% (1 955)	21.6% (1 861)	14.4% (94)
Middle occupation	26.7% (2 469)	27.0% (2 323)	22.4% (146)
Farmer and skilled blue-collar worker	3.4% (310)	3.3% (283)	4.1% (27)
Clerk	43.5% (4 028)	43.2% (3 714)	48.1% (314)
Manual worker, unemployed, student	5.3% (488)	4.8% (416)	11.0% (72)
Household income per consumption unit (€/month)	1619 (1238–2048)	1667 (1250–2056)	1429 (1062–1833)
Missing values	523	468	55

Data are median (Interquartile range) unless otherwise indicated.

Differences between individuals included in and excluded from the study are reported in Supplementary Table 1. Mothers excluded from the sample were more often single as compared with those included. As compared with excluded mothers, included mothers were more often descendants of immigrants or immigrant themselves; more frequently had ≤ high school education; more often belonged to the clerk or manual worker or unemployed and student occupational categories; and had a lower median household income.

**Figure 3:**
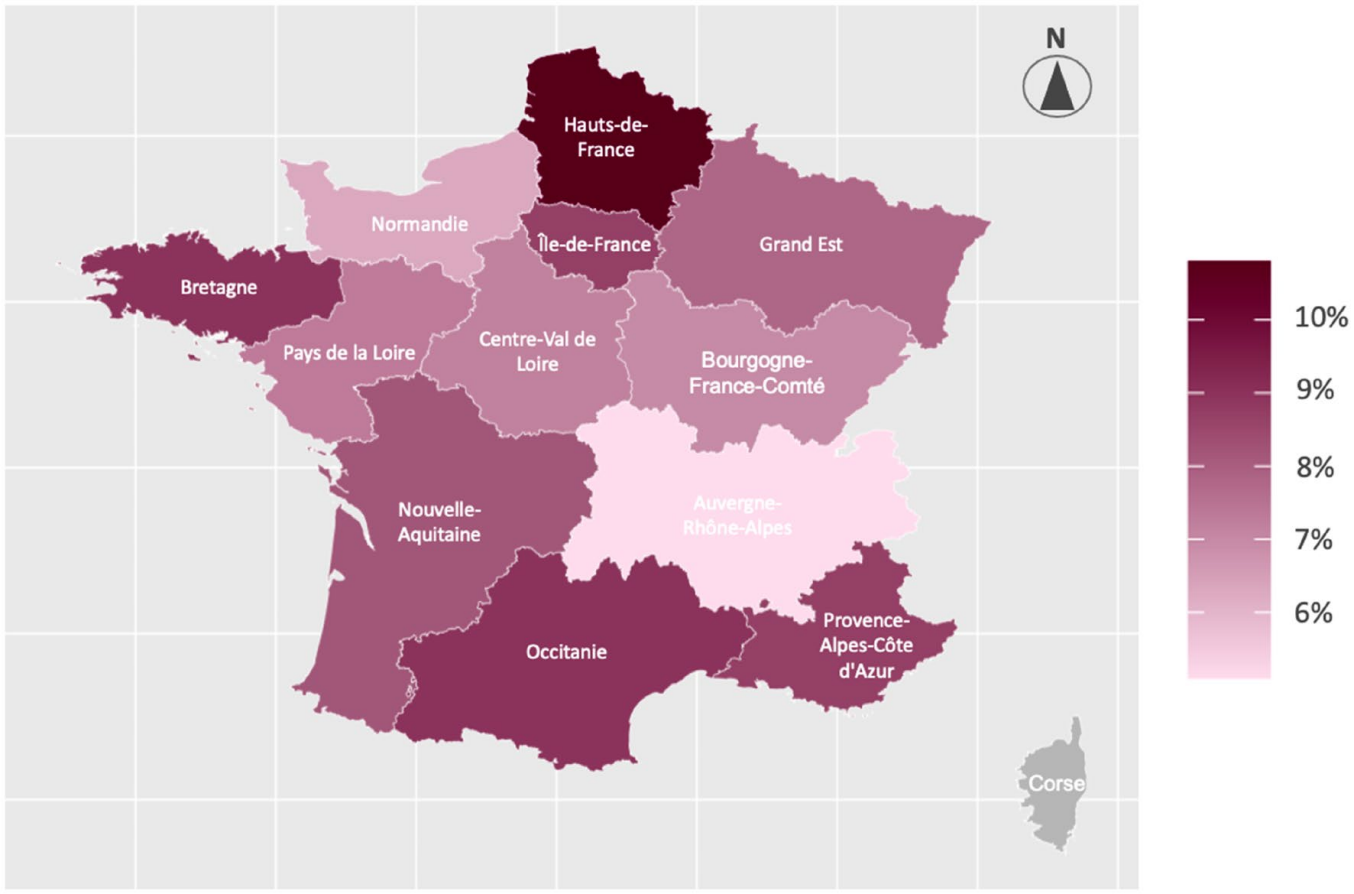
Weighted prevalence of overweight status in children aged 3.5 years from the ELFE cohort according to metropolitan France regions (n = 9238 children). *To ensure the validity of this regional prevalence analysis, we excluded children from the Corsica region (n = 12) because of insufficient sample size. **The map was generated on R version 4.2.1 (2022-06-23) using the sf package (v1.0.12) with shapefile downloaded from https://www.data.gouv.fr/en/datasets/contours-des-regions-francaises-sur-openstreetmap.

Of 9250 children included, 653 were classified as overweight: 7.1% (95% CI 6.5–7.6) before data weighting and 8.3% (7.7–9.0) after weighting (Table 1). The prevalence of overweight differed by regions, with the highest in Hauts-de-France (north) (10.8%) and the lowest in Auvergne-Rhône-Alpes (middle east) (5.1%; weighted prevalence) (Fig. 3).

**Figure 4:**
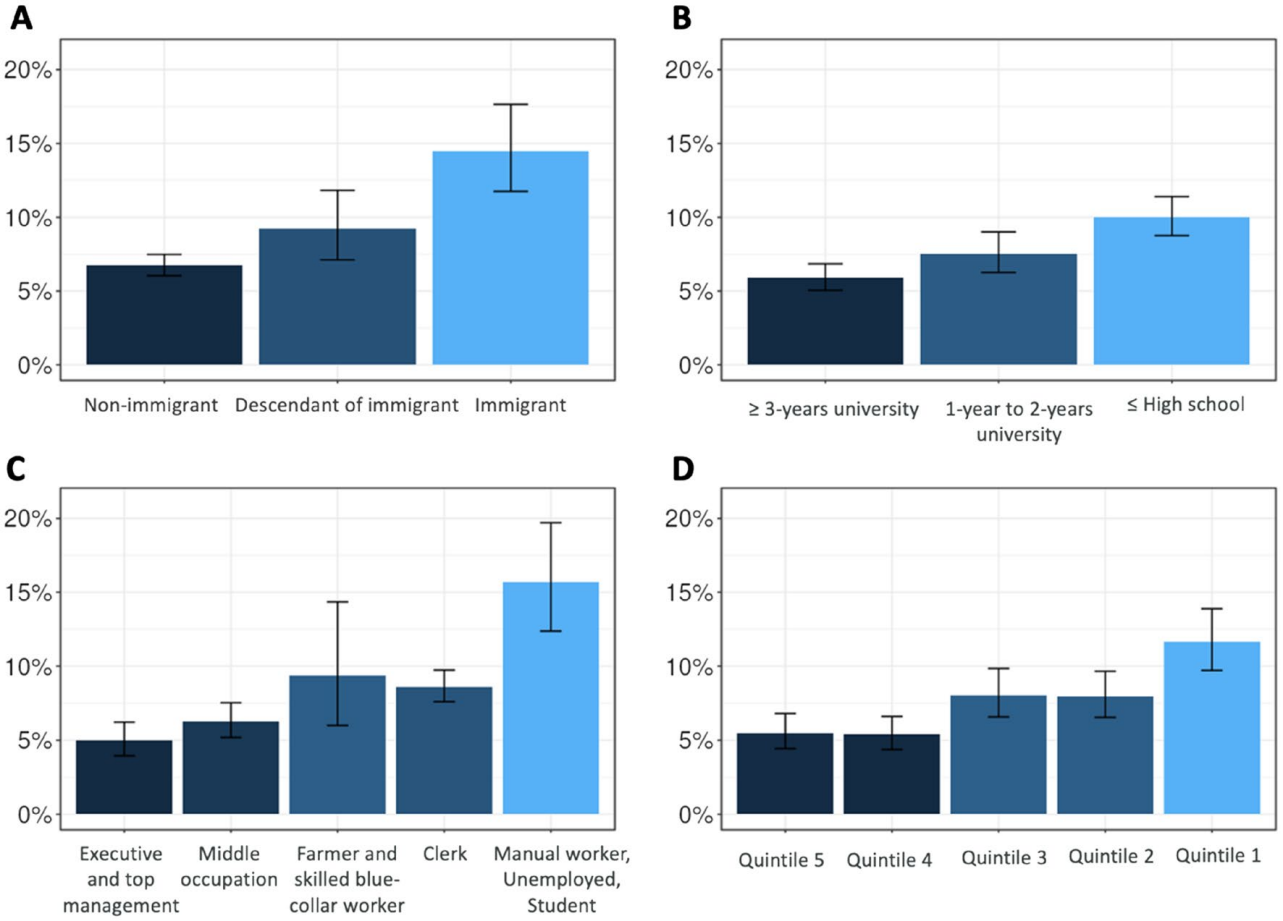
Weighted prevalence (95% CI) of overweight status according to maternal migration status and socioeconomic position indicators for children aged 3.5 years in the ELFE national cohort. (**A**) Maternal migration status (n = 8998); (**B**) maternal education level (n = 8928); (**C**) maternal occupational status (n = 9250); (**D**) quintiles of household income per consumption unit (n = 8727).

We found a social gradient in overweight for both migration and SEP (Fig. 4). The subgroups of children the most affected by excess weight were those born to immigrant mothers (weighted prevalence:14.4% [95% CI 11.7–17.6]), mothers with ≤ high school education (10.0% [8.8–11.4]); and mothers who were manual workers, unemployed, or students (15.7% [12.4–19.7]); and children living in households in the lowest income quintile (quintile 1) (11.6% [9.7–13.9]). For this final variable, the prevalence of overweight was intermediate for children from households in income quintile 2 and 3 (8.0% [95% CI 6.5–9.7] and 8.1% [6.6–9.8]). Given that we found no interaction between migration status and SEP ((p > 0.2), we did not stratify the analyses afterward.

In the multivariable models, the odds of overweight status differed by migration status and was inversely associated with all three dimensions of SEP (Table 2): the odds of overweight was increased for children whose mothers were immigrants or descendants of immigrants (OR 2.22 [95% CI 1.75–2.78] and 1.35 [1.04–1.74]) versus non-immigrant mothers; mothers with ≤ high school education or 2-year university degree (1.72 [1.42–2.08] and 1.36 [1.09–1.70]) versus ≥ 3-year university degree; and mothers in the manual worker, unemployed and student category or clerk category (2.54 [1.74–3.70] and 1.35 [1.02–1.78]) versus the executive and top management category. Regarding household income, the odds of overweight was increased for children in the lowest quintile (1.39 [1.02–1.91]) than the highest quintile.

**Table 2:** Unweighted multivariate associations between overweight status and socioeconomic position and maternal migration status for children aged 3.5 years in the ELFE national birth cohort (imputed data, n = 9 250).

Characteristics	Model 1			Model 2			Model 3			Model 4		
	OR	95% CI	p-value	OR	95% CI	p-value	OR	95% CI	p-value	OR	95% CI	p-value
Maternal migration status			< 0.001			< 0.001			< 0.001			< 0.001
Non-immigrant	–	–		–	–		–	–		–	–	
Descendant of immigrant	1.35	1.04–1.74		1.31	1.01–1.69		1.32	1.01–1.70		1.30	1.00–1.68	
Immigrant	2.22	1.75–2.78		2.17	1.71–2.72		2.02	1.59–2.54		1.90	1.49–2.41	
Maternal education level						** < 0.001**			**0.019**			0.10
≥ 3-year university				–	–		–	–		–	–	
1- to 2-year university				1.36	1.09–1.70		1.25	0.99–1.58		1.23	0.97–1.55	
≤ High school				1.72	1.42–2.08		1.38	1.10–1.74		1.28	1.01–1.63	
Maternal occupational category									** < 0.001**			**0.001**
Executive and top management							–	–		–	–	
Middle occupation							1.15	0.87–1.52		1.12	0.84–1.50	
Farmer and skilled blue collar worker							1.55	0.96–2.43		1.42	0.87–2.25	
Clerk							1.35	1.02–1.78		1.26	0.94–1.70	
Manual worker, unemployed, student							2.54	1.74–3.70		2.24	1.51–3.32	
Income per consumption unit												0.087
Quintile 5										–	–	
Quintile 4										0.94	0.72–1.24	
Quintile 3										1.18	0.88–1.58	
Quintile 2										1.18	0.87–1.58	
Quintile 1										1.39	1.02–1.91	

Results adjusted on the sex of the child, age of the mother and parity.

Significant values are in bold.

*OR* odds ratio, *CI* confidence interval.

*Results adjusted on the sex of the child, age of the mother and parity. According to the four–nested-variables logistic regression method, the results of each variable are assessed when it is first introduced in the model.

## Discussion

This study confirms a social gradient of overweight in children according to the mother’s educational level. However, the gradient was not as clear for maternal occupation and household income, given that it was rather a threshold effect contrasting the lowest SEP categories to the others, namely, “manual worker, unemployed and student” for occupation and the lowest quintile of income. Additionally, the mother’s migration status seemed to play an important role, without interacting with other SEP variables. We believe our analysis adds value to the existing literature by taking a holistic approach because it incorporates the perspective of migration history, thus better disentangling the multifaceted socio-cultural determinants contributing to this health condition.

The overall prevalence of overweight in our sample (8.3%, obesity included) was relatively low as compared with national estimates reported in three other studies. The INCA3 surveys, conducted in 2014 in metropolitan France and using IOTF cut-offs, estimated a prevalence of 13.7% in children 4 to 6 years old^25^. Notably, children participating in the ELFE cohort study were younger than these children, precisely 3.5 years old in 2014. Likewise, a nationwide representative school survey conducted in 2013 reported an overweight rate in kindergarten (mean age 5 years) of 11.9% (90% CI 11.5–12.5)^26^; these latter figures apply to all regions of France, including the overseas departments (except Mayotte), where the prevalence is much higher^27^. Both studies share a cross-sectional design, which could explain the difference in prevalence with the ELFE cohort, more likely to be affected by selection and attrition biases (as is the case in most cohorts) that can only partly be corrected by weighting. Nevertheless, our findings are consistent with the inverse socio-economic gradient reported from pre-school age in these two other national studies, whether the definition of SEP was based on the highest occupational category among the two parents^26^ or the educational level of the respondent parent in INCA3^25^. The latter study found no association with respondent occupational status. Although the territorial divide of childhood overweight has not yet been described in France, in adults, the Obepi survey reported a downward North–South gradient of obesity^28^. Here, we show that the geographical distribution is slightly different in early childhood, with the Northern, Southern and Western regions most affected.

The overall inverse socio-economic gradient of overweight observed in the 3.5-year-old children of the ELFE cohort did not apply uniformly to all SEP dimensions. Our findings show that for household income levels, children in the lowest quintile but not the intermediate ones were more likely to have overweight than those in the highest income quintile. Likewise, children born to “workers, unemployed and student” mothers only were more likely to have overweight than children born to mothers in the reference occupational category. This lack of clear gradient for occupational status can be partly explained by some categories being quite heterogenous, especially “farmer and skilled blue-collar worker” and “manual worker, unemployed, student,” but we could not maintain a finer granularity because of the sample size. More generally, occupation-based indicators are most often non-hierarchical^29^. Still, all SEP indicators, although interdependent, reflect different facets of the SEP and are not equally stable over time. For instance, income is known to be more volatile, and external shocks can temporarily impact income levels without necessarily altering household conditions. Furthermore, in our study, although a continuous gradient seems to apply to maternal education, the effects of income and occupation seem to be subject to a threshold beyond which they are no longer associated with child overweight. These two different paradigms^30^ have implications for interventions aimed at reducing health inequalities in terms of components and sub-population groups to be targeted in priority^30^. They can influence the adoption of proportionate universalism, a perspective that seeks to provide universal access to health services and interventions while directing resources to those who are most in need: this approach recognises that everyone should have access to the same level of basic care but acknowledges that some individuals and communities require additional support to achieve optimal health outcomes^30–32^.

Furthermore, our results show that the association between maternal education and child overweight was substantially attenuated after adjusting for household income. This observation suggests that the effect of maternal education on child overweight in the ELFE cohort is partly explained by household income, as was further confirmed by a mediation analysis (results not shown, but available on request). However, there is some remaining direct effect of education, as confirmed by other studies^7,33,34^: for example Van Rossem et al.^33^ reported a persistent but attenuated association between maternal educational level and child overweight after adjusting for material hardship in the Dutch Generation R cohort.

To our knowledge, our study is the first to investigate the association between maternal migration status and childhood overweight in France. The results are consistent with a systematic review of 19 studies conducted in six European countries (not France) published from 1999 to 2009 that overall found a higher risk of overweight and obesity in immigrant children than their non-immigrant counterparts^11^. However, most of these studies did not control for socio-economic factors in their analyses and the definition used to classify a child as an “immigrant” was not consistent across all studies. Other research from the Generation R study showed that children whose parents were born abroad were more likely to be overweight at age 4 years than children whose parents were both born in The Netherlands. However, after adjusting for maternal education, parental BMI, and infant weight change, the associations were attenuated, with the strongest attenuation observed after adjustment for maternal education^33^.

The “healthy migrant effect” describes migrants having better health outcomes than non-migrants in both their source and host countries. Evidence for the healthy migrant effect is inconsistent across regions and contexts. Previous studies^15,35^ have documented the healthy migrant effect in Canada, the United States, Australia and the United Kingdom for the prevalence of chronic conditions, self-assessed health, and obesity, but the situation in other European countries is complex and heterogeneous. Moullan et al.^14^ found evidence of a healthy migrant effect for self-assessed health in Italy and Spain but not Belgium and France. Some other studies have reported poorer health status among immigrants than non-immigrants in terms of chronic conditions and self-perceived health in France^16,17,36^ and Europe^37^. Differences between countries can be explained by factors such as the country of origin, the host country’s legal framework for immigration, the length of stay, and the healthcare system; the healthy migrant effect also depends on the reasons for migration^15^. Whether this effect extends to the offspring of immigrant mothers with regard to obesity remains unclear. A US study did not find any “healthy foreign-born effect” for childhood obesity^38^.

A recent study based on the ELFE cohort showed that immigrant parents’ pre-migration education had a positive impact on their children’s birth outcomes including birth weight^39^, but this advantage declined with length of residence in the new country. The acculturation process, which refers to the change and adaptation that occurs when individuals or groups encounter a different culture, may partly account for why this benefit diminishes with length of residence. This process entails changes in attitudes, values, beliefs, behaviors and language and can take place at both individual and group levels. A systematic review concluded that acculturation was associated with obesity in adult immigrants from low/middle-income to high-income countries^40^. Another Swedish study found that children of immigrants had greater risk of low physical activity and overweight than children of Swedish parents, despite a better-quality diet^15^. However, acculturation is a complex and nuanced phenomenon that requires careful examination^41^. Another study of ELFE data found that the diet of immigrant mothers was better than that of descendants of immigrants, who in turn had a better diet than non-immigrant mothers^42^. Therefore, women who were less acculturated had both a healthier and less processed diet than non-immigrant mothers^43^. However, this relationship must be tested in children to determine the extent to which it applies to them. In light of the literature^42,44^, we were expecting a more pronounced socio-economic gradient of childhood overweight in the non-immigrant than immigrant population, which was not confirmed given the lack of interaction between migration status and all three SEP indicators.

In the present analysis, the association between maternal migration status and childhood overweight persisted even after adjusting for other SEP variables. This finding suggests that migration status has a direct effect, independent of SEP. In particular, socio-cultural norms, values and representations could be involved. For example, perception of weight and overweight may differ according to the migration status and induce less favorable energy balance-related behaviors, regardless of SEP: a British study found that parental perceptions of healthy body size and concerns about overweight in childhood varied by ethnicity^45^. Disparities observed between different racial and ethnic groups could also indicate discrimination at an institutional or structural level, shaped by personal experiences, or a combination of both^7^. However, we cannot rule out the possibility of residual confounding due to other unmeasured dimensions of SEP. For instance, a study^34^ of the ELFE cohort used a multidimensional approach to measure child poverty, combining income and deprivation measures. Income poverty did not perfectly overlap with deprivation: some low-income children were not considered poor and some higher-income children were considered deprived.

There are a number of limitations to our study. As mentioned before, the prevalence estimates reported in this cohort are lower than those from other cross-sectional studies. The specific weights provided by the ELFE team to adjust the sample and mitigate these selection and attrition biases were deemed effective but not sufficient to correct the selection bias due to attrition. However, our primary objective was not to measure the prevalence itself but rather to comprehend the social patterning of childhood overweight with a holistic approach, accounting for various social determinants, in particular migration. Still, the associations under study with the SEP and migration indicators may have been stronger with a better representation of the most socially disadvantaged families. Of note, the ELFE cohort does not include children from the overseas departments. A study published in 2012 on children aged 5 to 9 years in Guadeloupe, Martinique, French Guiana and French Polynesia reported an overweight prevalence of 15.2%, 25.0%, 16.8% and 31.6%, respectively^27^. Hence, this exclusion could potentially bias the results by underestimating the absolute prevalence and the prevalence in the non-immigrant population. In addition, several studies have reported ethnic variations in body composition^46–48^. For the purpose of consistency, we decided to use the most commonly accepted definition of overweight: BMI greater than or equal to the IOTF-25 cut-off. However, by extension, this choice may slightly overestimate the real prevalence of overweight among some ethnic minorities^7^.

Our study also has notable strengths. The use of a hierarchical approach in the design of our conceptual model avoids a wrong interpretation of over-adjustment commonly encountered in other studies that incorporate all variables simultaneously in the same models. In addition to confirming that “one size does not fit all”^7^, our approach demonstrated both social gradient and threshold effects. Finally, the use of multiple imputation techniques mitigated potential bias introduced by missing data, which provided a significant advantage.

This study demonstrates the existence of social inequalities in the ELFE birth cohort. These findings, based on SEP and migration status, agree with those of other studies conducted in similar European cohorts^11^, such as Generation R^33^. However, more nuanced aspects of vulnerability^34^ need to be examined to gain a deeper understanding of the underlying mechanisms that structure and mediate the effect of these factors on child overweight. This examination would enable the development of concrete solutions to be applied in public health interventions. Nonetheless, if the latter have the overarching goal to reduce social inequalities in childhood overweight, their design requires an appropriate balance between individual versus structural components. Indeed, a systematic review revealed that interventions across different socio-economic groups differed in efficacy^49^. Those that relied on information provision for individual behavior modification were ineffective in participants with lower SEP. Only community or policy interventions aimed at bringing structural changes to the environment were successful for them. This is also corroborated by a recent systematic review which highlighted that increasing the availability of healthier food options enhanced the likelihood of healthy choices and reduced the energy content of the diet similarly among individuals with higher and lower SEP^50^. There are existing examples of this type of intervention that have demonstrated positive outcomes in encouraging healthier choices among consumers^51^. Hence, policies that increase the availability of healthier food could have potential as equitable strategies to reduce obesity and improve population health. In addition to availability, accessibility is a crucial factor in food choices. Research suggests a significant positive effect of higher occupational social class on food expenditure, which in turn influences the healthiness of food purchases^52^. Within the framework of proportionate universalism, only such structural interventions can empower the segment of the population facing social adversity and promote social equity in health initiatives. Another recent systematic review found that interventions delivered by lay agents among ethnic/racial minorities had some effect on lifestyle behaviors and obesity risk; additional factors for creating more effective, pragmatic, inclusive, and non-judgmental programs were to engage stakeholders, including users, in their development and adhering to theoretical frameworks^53^.

## Conclusion

We confirmed the presence of social inequalities in childhood overweight in the ELFE cohort, appearing as early as 3.5 years. Mother’s migration status was a risk factor for child overweight, even after accounting for several SEP dimensions. Although our study corroborates the existence of a social gradient of overweight in children based on maternal educational level, this gradient was less evident for maternal occupation and household income, which indicates a threshold effect. Hence, some populations are more vulnerable than others, in particular children of mothers who are immigrants or immigrant descendants, those who are manual workers or unemployed, or with low income. Therefore, these factors should be considered for better social equity when designing obesity prevention interventions.

### Supplementary Information

Supplementary Information.

## Data Availability

The datasets analyzed during the current study is not publicly available due to ethical restrictions related to protection of participant confidentiality and legal restrictions imposed by the French National Commission on Data Processing and Liberties (CNIL). Investigators who wish to access the data reported in this article must address a reasonable request to the Elfe’s data access committee (CADE) at contact@elfe-france.fr.
