# Author Correction: Social patterning of childhood overweight in the French national ELFE cohort

**DOI:** 10.1038/s41598-024-53416-2

**Published:** 2024-02-06

**Authors:** Camille Le Gal, Marion Lecorguillé, Lorraine Poncet, Aminata Hallimat Cissé, Malamine Gassama, Thierry Simeon, Jean‑Louis Lanoë, Maria Melchior, Jonathan Y. Bernard, Marie‑Aline Charles, Barbara Heude, Sandrine Lioret

**Affiliations:** 1 Université Paris Cité and Université Sorbonne Paris Nord, Inserm, INRAE, Center for Research in Epidemiology and StatisticS (CRESS), 75004 Paris, France; 2 UMS INED-INSERM-EFS, Paris, France; 3 IPLESP, INSERM U1136, Paris, France

Correction to: *Scientific Reports* 10.1038/s41598-023-48431-8, published online 11 December 2023

The original version of this Article contained an error in Table 1, where the column headings were incorrect.

As a result,

“Not overweight N = 653 7.1%”

now reads:

“Not overweight N = 8 597 92.9%”

And,

“Overweight N = 8 597 92.9%”

now reads:

“Overweight N = 653 7.1%”

The original Article has been corrected.

