# Supplementary material for: Social patterning of childhood overweight in the French national ELFE cohort
Source: Sci Rep. 2023 Dec 11;13:21975. doi: 10.1038/s41598-023-48431-8 (PMC10713558; doi:10.1038/s41598-023-48431-8)
Supplement: Supplementary file 1 — Supplementary Information. [file 41598_2023_48431_MOESM1_ESM.pdf]

Supplementary Table 1: Comparison of included and excluded children aged 3.5 years in the ELFE national cohort (n = 18,329) (unweighted data)

	**Included** N = 9 250	**Excluded** N = 9 079	**p-value**[1]
**Sex of the child**			
Girl	48.9% (4 519)	48.3% (4 340)	0.5
**Maternal age at delivery** (years)	31.0 (28.0–35.0)	30.0 (26.0–34.0)	<0.001
**Parity**			<0.001
First-time mother	48.1% (4 386)	43.7% (3 863)	
Second-time mother	35.9% (3 279)	33.6% (2 968)	
Multiparous	16.0% (1 457)	22.7% (2 004)	
**Single motherhood**			<0.001
Yes	2.5% (221)	6.2% (462)	
**Maternal migration status**			<0.001
Non-immigrant	81.7% (7 355)	76.3% (5 650)	
Descendant of immigrant	9.8% (878)	11.4% (844)	
Immigrant	8.5% (765)	12.3% (915)	
**Maternal country of birth**			<0.001
France	90.3% (8 347)	83.5% (7 466)	
European Union	2.3% (213)	2.1% (188)	
Turkey and Maghreb	3.3% (301)	6.7% (600)	
Sub-Saharan Africa	2.4% (222)	5.0% (447)	
Other	1.8% (164)	2.7% (239)	
**Maternal education level**			<0.001
≥ 3-year university	43.5% (3 886)	27.2% (2 000)	
1- to 2-year university	24.0% (2 142)	19.7% (1 451)	
≤ High school	32.5% (2 900)	53.1% (3 902)	
**Maternal occupational category**			<0.001
Executive and top management	21.1% (1 955)	12.4% (1,115.0)	
Middle occupation	26.7% (2 469)	17.1% (1,545.0)	
Farmer and skilled blue-collar worker	3.4% (310)	4.0% (363.0)	
Clerk	43.5% (4 028)	50.2% (4,532.0)	
Manual worker, unemployed, student	5.3% (488)	16.2% (1 465)	
**Household income per consumption unit** (€/month)	1 619 (1 238–2 048)	1,389 (952–1,778)	<0.001
**Maternal BMI** (kg/m²)	22.1 (20.2–25.1)	22.4 (20.2–25.8)	<0.001
**Maternal weight status**			<0.001
Thin	7.2% (660)	8.5% (747)	
Normal	67.4% (6 143)	61.7% (5 421)	
Overweight	25.4% (2 315)	29.8% (2 614)	
**Paternal BMI** (kg/m²)	24.3 (22.4–26.8)	24.5 (22.3–27.0)	0.7
**Paternal weight status**			>0.9
Thin	0.8% (6)	0.9% (3)	

Normal	57.0% (405)	56.0% (183)	
Overweight	42.2% (300)	43.1% (141)	
**Weight for gestational age**			0.001
Small-for-gestational-age	6.7% (610)	8.1% (716)	
Appropriate-for-gestational-age	88.3% (8 035)	87.1% (7 664)	
Large-for=gestational-age	5.0% (453)	4.8% (421)	
**Type of delivery**			<0.001
Spontaneous delivery	67.4% (6 189)	67.2% (5 982)	
Forceps, spatulas, suction cups	13.3% (1 219)	11.4% (1 017)	
Caesarean section	17.5% (1 603)	19.4% (1 724)	
Unknown	1.9% (170)	2.0% (175)	
**Gestational diabetes**			
Yes	7.1% (629)	8.0% (683)	0.029
**Smoking during pregnancy**			
Yes	16.9% (1 547)	23.5% (2 077)	<0.001

Data are median (IQR) unless otherwise indicated.

[1] Wilcoxon rank sum test; Pearson's chi-squared test; Fisher's exact test

Supplementary Table 2: Unweighted multivariable associations of overweight status and socio-economic position and maternal migration status for children aged 3.5 years in the ELFE national birth cohort (complete cases, n = 8 524)

	**Model 1**			**Model 2**			**Model 3**			**Model 4**		
**Characteristics**	**OR**	**95% CI**	**p-value**	**OR**	**95% CI**	**p-value**	**OR**	**95% CI**	**p-value**	**OR**	**95% CI**	**p-value**
**Maternal migration status**			<0.001			<0.001			<0.001			<0.001
Non-immigrant	—	—		—	—		—	—		—	—	
Descendant of immigrant	1.36	1.03–1.77		1.32	1.00–1.71		1.32	1.00–1.72		1.31	0.99–1.70	
Immigrant	2.25	1.76–2.86		2.20	1.71–2.81		2.04	1.58–2.60		1.93	1.49–2.49	
**Maternal education level**						<0.001			0.10			0.2
≥ 3-year university				—	—		—	—		—	—	
1-year to 2-year university				1.34	1.07–1.68		1.23	0.97–1.56		1.21	0.95–1.54	
≤ High school				1.61	1.31–1.97		1.29	1.01–1.65		1.19	0.93–1.54	
**Maternal occupational category**									<0.001			<0.001
Executive and top management							—	—		—	—	
Middle occupation							1.14	0.86–1.53		1.11	0.82–1.50	
Farmer and skilled blue-collar worker							1.45	0.86–2.34		1.34	0.79–2.19	
Clerk							1.35	1.01–1.81		1.28	0.94–1.74	
Manual worker, unemployed, student							2.84	1.87–4.30		2.54	1.64–3.92	
**Income per consumption unit**												0.2
Quintile 5										—	—	
Quintile 4										0.94	0.71–1.26	
Quintile 3										1.19	0.88–1.61	
Quintile 2										1.14	0.84–1.56	
Quintile 1										1.35	0.97–1.88	

[1] OR: odds ratio, CI: confidence interval, Results adjusted on the sex of the child, age of the mother and parity.
**Results adjusted on the sex of the child, age of the mother and parity. According to the four–nested-variables logistic regression method, the results of each variable are assessed when it is first introduced in the model.*
